# Spindle cell sarcoma: a SEER population-based analysis

**DOI:** 10.1038/s41598-018-23145-4

**Published:** 2018-03-22

**Authors:** Lei Feng, Meng Wang, Feiluore Yibulayin, Hao Zhang, Yin-Long Yang, Fei Ren, Alimujiang Wushou

**Affiliations:** 1 Department of Oral and Maxillofacial Surgery, Shanghai Stomatological Hospital, Fudan University, Shanghai, 200001 China; 2 Oral Biomedical Engineering Laboratory, Shanghai Stomatological Hospital, Fudan University, Shanghai, 200001 China; 3 Department of Epidemiology and Biostatistics, Shanghai Stomatological Hospital, Fudan University, Shanghai, 200001 China; 4 Department of preventive medicine, school of public health, Fudan University, Shanghai, 200001 China; 5 Department of Oncology, Shanghai Medical College, Fudan University, Shanghai, 200001 China; 6 Department of Pathology, Fudan University Shanghai Cancer Center, Shanghai, 200032 China

## Abstract

Due to the substantial limitation of study population, Spindle cell sarcoma (SCS) was unexplored comprehensively. In this study, we investigated the clinical characteristics and disease specific prognostic factors of SCS. 3299 SCS cases were identified and extracted from Surveillance, Epidemiology, and End Results (SEER) database (1973–2017). White people account for 79.1% with median age of 57 years without predominance in any gender. Significant disease specific survival (DSS) and overall survival (OS) were found differentiated in age, T stage, N stage, M stage, AJCC stage, SEER historic stage, tumor locations, surgery, and pathologic grade. In the multivariate Cox analysis, the age >64 years (for DSS, P < 0.001 and for OS, P < 0.001; Reference age ≤64 years), AJCC stage III (for DSS, P = 0.006 and for OS, P = 0.04; Reference: AJCC stage I), and non-surgical treatment (for DSS, P < 0.001 and for OS, P < 0.001; Reference: surgery) were independently associated with worse DSS and OS. In brief, our study demonstrated that SCS mostly found in white people at fifth to seventh decades of life without gender predilection. The patient’s age, AJCC stage, tumor location and surgery were independent prognostic indicators for both DSS and OS of SCS.

## Introduction

Spindle cell neoplasm are diverse in nature by means of clinicopathologic and tumor biological heterogeneity^1^. Primary spindle cell sarcoma (SCS) is an extremely rare entity and one of the least reported tumor^2^. It is a type of connective tissue tumor and generally begins in layers of connective tissue such as that under the skin, between muscles, and surrounding organs. Only a handful of cases have been reported around the world from variety of body parts^3–9^. As such, SCS constitutes a diagnostic and therapeutic challenge^10,11^.

As morbidity, majority of the previous reports were single case reports and retrospective case series with more than five patients were even few. According to these case reports, the clinical presentations of SCS were similar to the benign lesion at early stage^11–18^. Like other sarcoma, SCS were treated aggressively with surgical therapy as a mainstay in the management and adjuvant (chemo) radiotherapy was implemented for patients with high risk behavior^2,5,7,9,10,13,15–18^.

Nowadays, the sophisticated molecular pathologic diagnostic techniques has made the diagnosis of SCS accurate and reliable^2^. However, owing to the rarity of SCS, there are lack of basic information regarding the tumor incidence, distinctive clinical characteristics, treatment outcome and disease specific prognostic factors. To address these, a retrospective investigation was carried out with study population from Surveillance, Epidemiology, and End Results (SEER) database.

## Results

### Summary statistics

**Figure 1:**
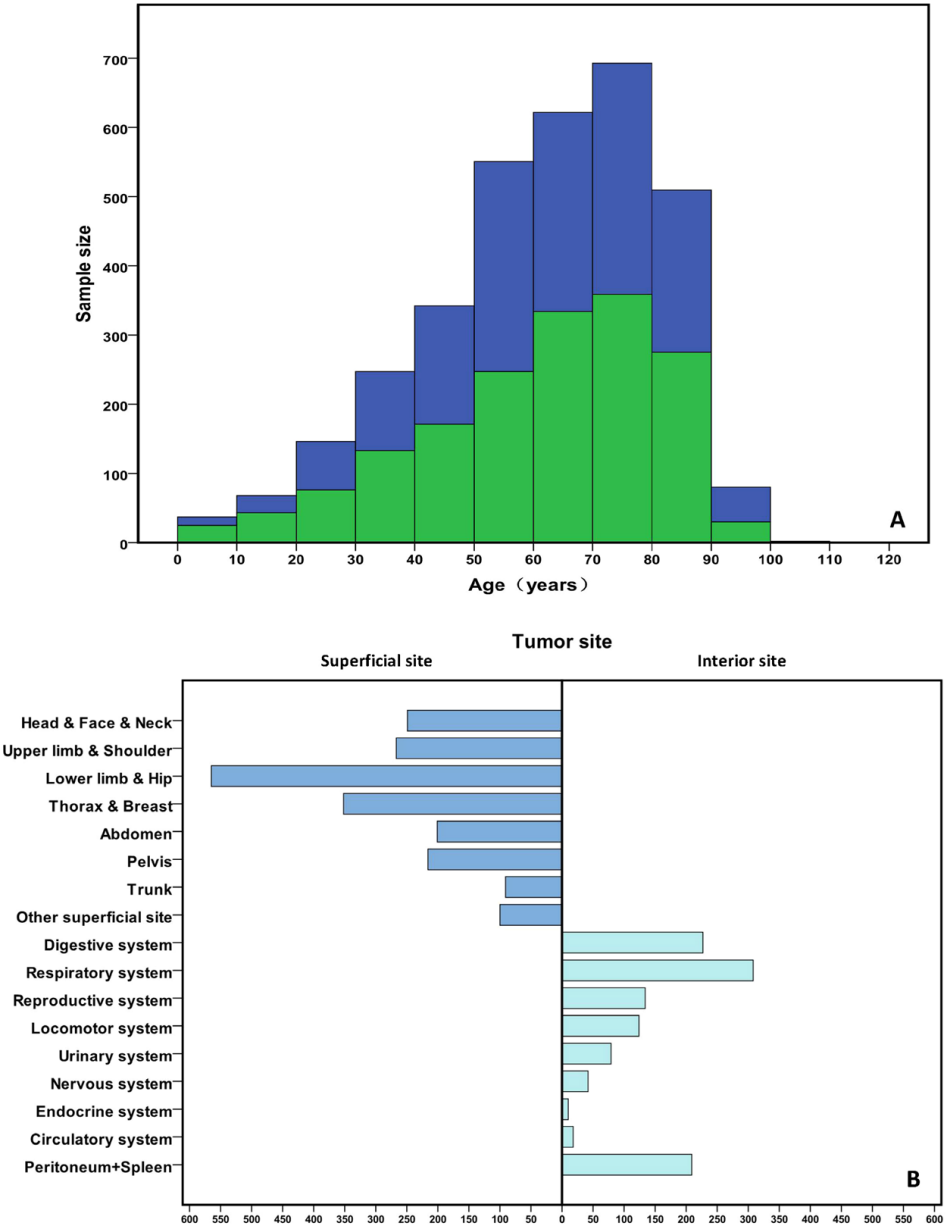
The distribution of age (**A**) and primary tumor site (**B**) of all SCS cases.

A total of 3299 cases were identified. The average follow-up time was 43 months (SD = 65), with the median follow-up time was 15 months (range, 1–481 months). Of these patients, the mean age at diagnosis of patients was 61 years (SD = 19, range from birth to 103 years) (Fig. 1). The incidence peaked during the seventh decade of life and the majority of cases were white people (80.9%, 2668/3299). There is no significant difference for gender distribution including 1605 females and 1694 males. According to this dataset, SCS could occur at almost any site of the body. The incidence was higher in superficial primary tumor site than internal primary tumor site (2041 vs. 1151 cases). More than 60% of overall case were treated surgery alone.

Among 3229 cases, 2115 cases were found with SCS specific mortality, in which included 1026 female and 1089 male with median age was 57 years (SD = 19). In this disease specific survival (DSS) group, white people account for nearly 80% of population (79.1%, 1674/2115). Regarding the pathological diagnosis, poorly differentiated cases were 27.5% (357/1300) and undifferentiated cases were 42.6% (554/1300). There were 302 early stage cases (AJCC stage I + II) and 367 advanced stage cases (AJCC stage III + IV). The basic clinic-pathological characteristics of overall study population and DSS subgroup summarized in Table 1.

**Table 1:** Epidemiological and clinico-pathologic characteristics of SCS patients.

Characteristic	Disease specific survival				Overall survival			
	Alive	Dead	Total	*P* value	Alive	Dead	Total	*P* value
Gender				0.270				0.088
Female	416	610	1026		510	1095	1605	
Male	416	673	1089		492	1202	1694	
Age				<0.001				<0.001
≤64	620	665	1285		705	1000	1705	
>64	212	618	830		297	1297	1594	
Race				<0.001				<0.001
White	653	1021	1674		794	1874	2668	
Black	86	171	257		105	270	375	
Others	74	89	163		84	146	230	
Pathologic grade				<0.001				<0.001
I	66	29	95		72	63	135	
II	193	101	294		220	212	432	
III	126	231	357		161	385	546	
IV	195	359	554		243	630	873	
AJCC Stage				<0.001				<0.001
I	136	29	165		171	74	245	
II	110	27	137		146	69	215	
III	86	86	172		105	158	263	
IV	23	172	195		35	267	302	
T stage				<0.001				<0.001
Tx + T0	90	188	278		116	333	449	
T1	162	39	201		218	112	330	
T2	228	250	478		269	424	693	
T3	4	16	20		5	24	29	
T4	2	16	18		2	28	30	
N stage				<0.001				<0.001
Nx + N0	474	458	932		591	841	1432	
N1	12	44	56		19	72	91	
N2	0	7	7		0	8	8	
M stage				<0.001				<0.001
Mx + M0	465	314	779		581	627	1208	
M1	21	195	216		29	294	323	
SEER historic stage				<0.001				<0.001
Localized	512	258	770		622	649	1271	
Regional	209	311	520		246	547	793	
Distant	43	482	525		53	722	775	
Marital status			0.972				0.238	
Married	428	659	1087		526	1195	1721	
Others	404	624	1028		476	1102	1578	
Surgery				<0.001				<0.001
Performed	703	577	1280		845	1172	2017	
Not performed	109	680	789		135	1078	1213	
Site				<0.001				<0.001
Internal	196	551	747		229	922	1151	
Superficial	629	658	1287		764	1277	2041	

### Survival analysis

**Figure 2:**
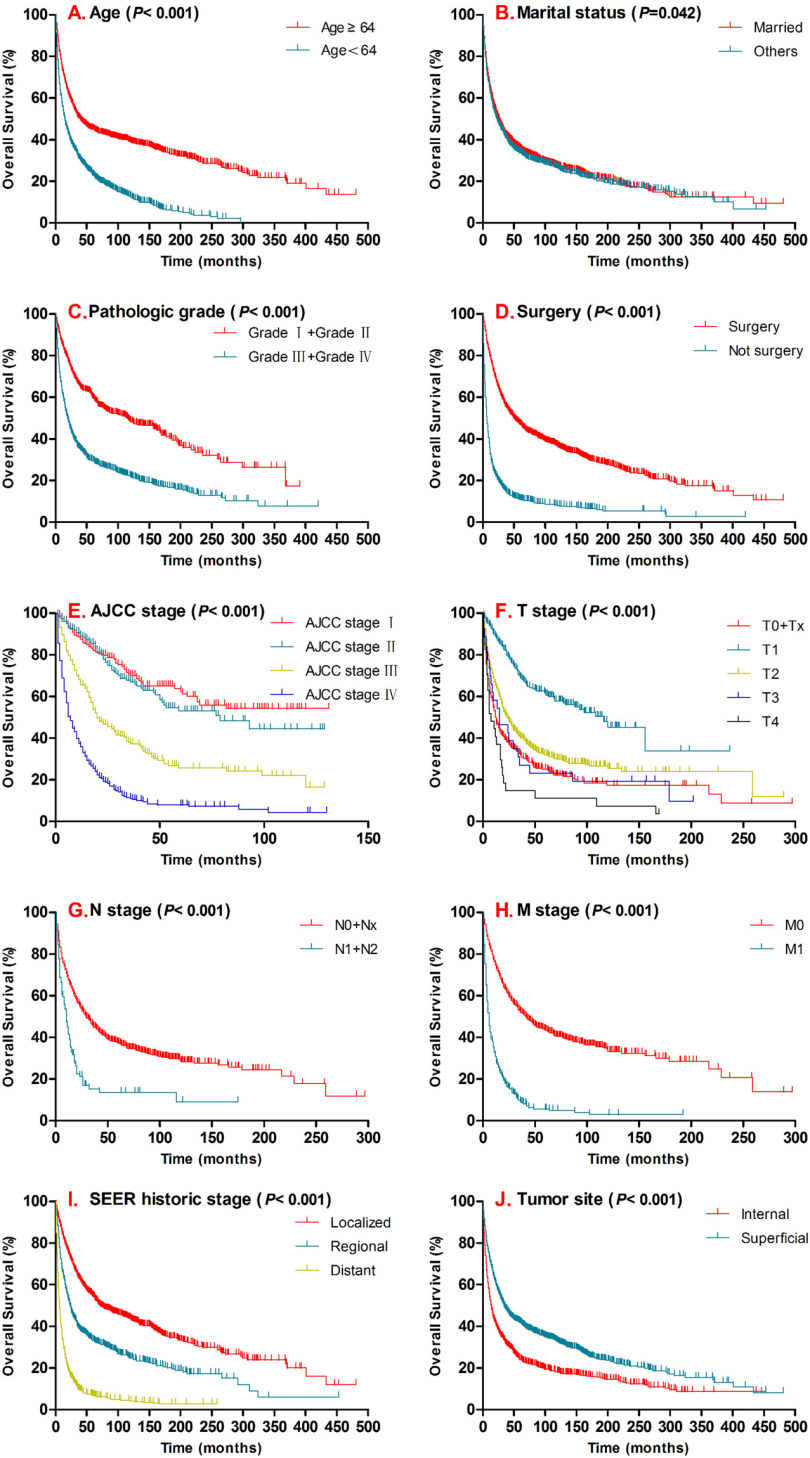
Kaplan–Meier curves for overall survival compared by age (**A**), marital status (**B**), pathologic grade (**C**), surgery (**D**), AJCC stage (**E**), T stage (**F**), N stage (**G**), M stage (**H**), SEER historic stage (**I**), tumor site (**J**). SEER data 1973–2017.

**Figure 3:**
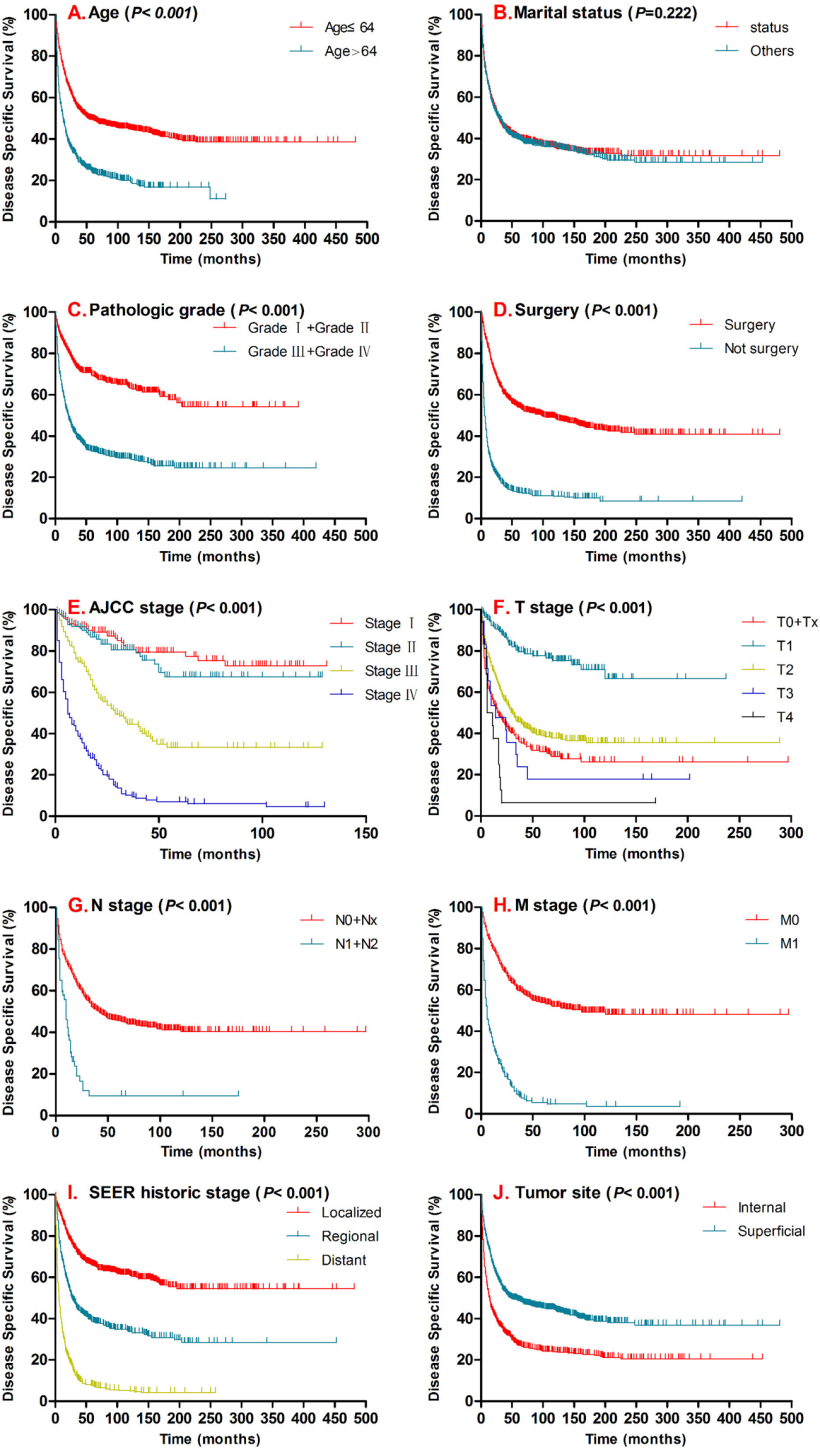
Kaplan–Meier curves for disease specific survival compared by age (**A**), marital status (**B**), pathologic grade (**C**), surgery (**D**), AJCC stage (**E**), T stage (**F**), N stage (**G**), M stage (**H**), SEER historic stage (**I**), tumor site (**J**). SEER data 1973–2017.

Survival analysis were performed as previously described^19,20^. There were significant differences depending on age (*P* < *0.001*), marital status (*P* = 0.042), pathologic grade (*P* < *0.001*), AJCC stage (*P* < *0.001*), T stage (*P* < *0.001*), N stage (*P* < *0.001*), M stage (*P* < *0.001*), SEER historic stage (*P* < *0.001*), tumor site (*P* < *0.001*) and treatment modality (*P* < *0.001*) in overall survival (OS) **(**Fig. 2). While, the significant differences were also found DSS for age (*P* < *0.001*), pathologic grade (*P* < *0.001*), AJCC stage (*P* < *0.001*), T stage (*P* < *0.001*), N stage (*P* < *0.001*), M stage (*P* < *0.001*), SEER historic stage (*P* < *0.001*), tumor site (*P* < *0.001*) and treatment modality (*P* < *0.001*) (Fig. 3).

In the univariate cox regression analysis, age, race, pathologic grade, AJCC stage, T stage, N stage, M stage, SEER historic and stage treatment were associated with DSS and OS (Table 2). More importantly, the age >64 years [HR 95% CI: 2.149 (1.619–2.851), *P* < *0.001*, age ≤64 years – as Ref], AJCC stage III [HR 95% CI: 2.803 (1.352–5.813), *P* = 0.006, AJCC stage I – as Ref], and non-surgical treatment [HR 95% CI: 2.979 (2.154–4.120), *P* < *0.001*, surgery – as Ref] were independently associated with worse DSS. Besides, the age, marital status, AJCC stage, T stage, N stage, SEER historic stage, tumor location and treatment were also independently correlated with OS (Table 3).

**Table 2:** Univariate Cox proportional hazard regression models.

Characteristic	Disease specific survival		Overall survival	
	HR(95% CI)	*P* value	HR(95% CI)	*P* value
**Gender**				
Female	1.00 Reference		1.00 Reference	
Male	1.049(0.940–1.171)	0.392	1.054(0.971–1.145)	0.205
**Age**				
≤64	1.00 Reference		1.00 Reference	
>64	2.203(1.971–2.462)	<0.001	2.039(1.874–2.218)	<0.001
**Race**				
White	1.00 Reference		1.00 Reference	
Black	1.105(0.940–1.299)	0.228	1.036(0.912–1.178)	0.583
Others	0.793(0.640–0.982)	0.034	0.878(0.745–1.035)	0.122
**Pathologic grade**				
I	1.00 Reference		1.00 Reference	
II	1.136(0.752–1.717)	0.546	1.097(0.828–1.454)	0.517
III	3.028(2.056–4.458)	<0.001	2.342(1.793–3.060)	<0.001
IV	3.156(2.160–4.612)	<0.001	2.477(1.910–3.212)	<0.001
**AJCC Stage**				
I	1.00 Reference		1.00 Reference	
II	1.202(0.711–2.030)	0.492	1.103(0.795–1.532)	0.557
III	3.636(2.384–5.544)	<0.001	2.602(1.973–3.432)	<0.001
IV	11.170(7.494–16.649)	<0.001	6.379(4.912–8.284)	<0.001
**T stage**				
Tx + T0	1.00 Reference		1.00 Reference	
T1	0.187(0.132–0.264)	<0.001	0.289(0.233–0.359)	<0.001
T2	0.658(0.544–0.795)	<0.001	0.693(0.600–0.800)	<0.001
T3	1.103(0.662–1.837)	0.708	0.891(0.588–1.349)	0.584
T4	1.669(1.000–2.785)	0.050	1.387(0.943–2.041)	0.097
**N stage**				
Nx + N0	1.00 Reference		1.00 Reference	
N1	2.482(1.817–3.391)	<0.001	1.996(1.567–2.543)	<0.001
N2	3.554(1.680–7.518)	0.001	2.258(1.125–4.531)	0.022
**M stage**				
Mx + M0	1.00 Reference		1.00 Reference	
M1	4.575(3.802–5.507)	<0.001	3.707(3.211–4.279)	<0.001
**SEER historic stage**				
Localized	1.00 Reference		1.00 Reference	
Regional	2.249(1.906–2.654)	<0.001	1.759(1.569–1.972)	<0.001
Distant	6.532(5.587–7.636)	<0.001	4.755(4.255–5.312)	<0.001
**Marital status**				
Married	1.00 Reference		1.00 Reference	
Others	1.069(0.958–1.193)	0.230	1.087(1.002–1.180)	0.045
**Surgery**				
Performed	1.00 Reference		1.00 Reference	
Not performed	4.242(3.783–4.758)	<0.001	3.533(3.243–3.850)	<0.001
**Site**				
Internal	1.00 Reference		1.00 Reference	
Superficial	0.552(0.492–0.618)	<0.001	0.630(0.579–0.686)	<0.001

**Table 3:** Multivariate Cox proportional hazard regression models.

Characteristic	Disease specific survival		Overall survival	
	HR(95% CI)	*P* value	HR(95% CI)	*P* value
**Age**				
≤64	1.00 Reference		1.00 Reference	
>64	2.149(1.619–2.851)	<0.001	2.133(1.737–2.618)	<0.001
**AJCC Stage**				
I	1.00 Reference		1.00 Reference	
III	2.803(1.352–5.813)	0.006	1.662(1.023–2.699)	0.040
**T stage**				
Tx + T0	—	—	1.00 Reference	
T1	—	—	0.447(0.283–0.705)	0.001
T2	—	—	0.671(0.484–0.931)	0.017
**N stage**				
Nx + N0	—	—	1.00 Reference	
N1	—	—	1.557(1.049–2.312)	0.028
**SEER historic stage**				
Localized	1.00 Reference		1.00 Reference	
Regional	1.588(1.082–2.330)	0.018	—	—
Distant	4.020(1.590–10.164)	0.003	3.347(1.584–7.071)	0.002
**Marital status**				
Married	—	—	1.00 Reference	
Others	—	—	1.242(1.015–1.521)	0.035
**Surgery**				
Performed	1.00 Reference		1.00 Reference	
Not performed	2.979(2.154–4.120)	<0.001	2.810(2.215–3.565)	<0.001
**Site**				
Internal	1.00 Reference		1.00 Reference	
Superficial	0.473(0.333–0.673)	<0.001	0.552(0.427–0.715)	<0.001

## Discussion

According to current investigation, SCS affects people of almost all ages which was as same as soft tissue sarcomas^21^. SCS occur more commonly in middle and old age adult groups. In this series, SCS most frequently occurs during the seventh decades of life with the mean age at diagnosis of SCS is 61 years. In addition, there is no statistically significant difference on incidence rate in gender. However, there is predominance in male with a sex predilection of 1.11:1 male: female ratio in a previous report^22^. Besides, the overall race distribution includes 80.9% white, 11.4% black, 7.8% American Indian/Asian/Pacific Islander (Table 1). According to the survival analyses depending on demographic factors such as age, gender and race, it demonstrates that only age is an independent prognostic indicator for SCS in DSS and OS.

The treatment modalities were performed for SCS varied, including surgery, adjuvant radiotherapy and chemotherapy in previous available reports. In this study, we only concentrate on the obtainable treatment modality (surgery or not) and hopefully to confirm the role of surgery in SCS treatment. Despite of the difference in surgical style, the surgery group have absolute favorable survival in DSS and OS than non-surgery group. Thus, it indicates that surgical resection remains the mainstay of treatment for SCS. However, the value of extensive radical operation and lymphadenectomy is still ambiguous. Similarly, the descriptive results should not be misinterpreted as causal effects of surgery on survival because of the unavoidable severe treatment selection bias present in this retrospective data source. In addition, the use of adjuvant radiotherapy for SCS remains controversial, and the sensitivity of SCS to chemotherapy in the metastatic setting is highly variable^23^. Unfortunately, due to the lack of information on other therapies in this study, we are unable to determine the conclusion from this data that SCS patients cannot benefit from radiotherapy or chemotherapy.

The pathologic grade and TNM/AJCC stage are associated with outcome of sarcomas and it is important for treatment protocol planning^24–26^. In this study, although the pathologic grading data in this study was incomplete and half of them were missing in the SEER database, there are still 1986 cases available. According to the SEER Program user’s instruction, cases were listed with latest pathological grading system. Although two histological grading systems are mainly used for soft-tissue sarcoma: the National Cancer Institute (NCI) system and the French Federation of Cancer Centers Sarcoma Group (FNCLCC) system, but there is still no specific system can be used for spindle cell sarcoma. So we used the four-tiered grading system which was most commonly used, and recommended by the American Joint Commission on Cancer (National Cancer Institute, “Tumor Grade”, accessed 18 August, 2014)^27^. SCS were divided into four different pathologic grades basing on the degree of the cell differentiation^28^. In results, most of the cases are advanced grade at the first time when they are diagnosed, which includes 546 cases at grade III (pathologically poorly differentiated, 27.5%) and 873 cares at grade IV (pathologically undifferentiated, 42.6%). Previous reports demonstrated pathologic grade is a significant prognostic factor for outcome in soft tissue sarcomas^29^. Similarly the typical survival differences are found in pathologic grade for both DSS and OS (Figs 2C and 3C). Meanwhile, for TNM stage/AJCC stage survival analysis relatively complete data are available, including 1531cases for TNM staging data 1025 cases AJCC staging. By performing survival analysis, the significant survival difference in OS and DSS have been presented in T stage, N stage, M stage and AJCC stage (Figs 2E–H and 3E–H). Importantly, AJCC stage is one of the independent prognostic factors for SCS in DSS. Similarly, we confirmed SEER historic stage was another independent prognostic indicator for SCS patients. In this results, the SEER stage of distant metastasized tumor was unfavorably associated with DSS and OS for SCS (Localized tumor - as a ref).

The tumor origination is another important factor affecting the outcome of the tumor. SCS can occur in any anatomic location including soft tissue, bone, or viscera^30^. This study included all of the cases listed as spindle cell sarcoma which were pathologically confirmed (International Classification of Diseases for Oncology, Third Edition, Histologic Type ICD-O-3: 8801) including bone origination, meanwhile excluded undifferentiated high-grade pleomorphic sarcoma (8830/3) which is new category recognizes pleomorphic sarcomas that cannot be classified into any of the other categories. Above all, our study is the largest series of patients and intend to evaluate the primary tumor location as a prognostic factor for the first time. As previous studies, SCS occurred at any location of the body involving skin and subcutaneous connective tissue, tongue, sinus, trachea, atrium, vein, bone, etc.^11,31,32^. For better characterization and further evaluating, we categorized the tumor locations into two main groups according to the distribution of primary tumor site: superficial site (tumor involving skin and subcutaneous soft tissue in head & neck, upper limb & shoulder, lower limb & hip, thorax & breast, abdomen, pelvis, trunk and other) and interior site (included tumor involving bone or viscera of digestive system, respiratory system, reproductive system, locomotors system, urinary system, nervous system, endocrine system, circulatory system). In this categorization (Tables 4 and 5), we found that SCS was more likely to occur in the superficial site compare with the deep interior site (2014 superficial site cases versus 1151 interior site cases). And significant survival differences were found in both DSS and OS for SCS (Figs 2J and 3J). More importantly, tumor site is another independent prognostic indicator for SCS in both DSS and OS which means primary SCS locates in superficial site possibly have a better outcome.

**Table 4:** The distribution characteristics of SCS.

Characteristic	Disease specific survival				Overall survival			
	Alive	Dead	Total	*P* value	Alive	Dead	Total	*P* value
Superficial site (Subcutaneous, other soft tissue)				<0.001				<0.001
Head & Face & Neck	73	49	122		90	159	249	
Upper limb & Shoulder	121	59	180		144	123	267	
Lower limb & Hip	224	163	387		255	310	565	
Thorax & Breast	92	128	220		133	219	352	
Abdomen	36	97	133		39	162	201	
Pelvis	51	76	127		61	155	216	
Trunk	24	32	56		30	61	91	
Overlap and Other	8	54	62		12	88	100	
Internal site (Includes bone or viscera *et al*.)				0.007				0.003
Digestive system	42	99	141		49	178	227	
Respiratory system	50	151	201		60	248	308	
Reproductive system	27	66	93		28	106	134	
Locomotor system	29	40	69		38	86	124	
Urinary system	12	42	54		16	63	79	
Nervous system	9	16	25		9	33	42	
Endocrine system	2	4	6		4	6	10	
Circulatory system	2	12	14		2	16	18	
Peritoneum& Spleen	23	121	144		23	186	209

**Table 5:** Univariate Cox proportional hazard regression models of tumor sites.

Characteristic	Disease specific survival		Overall survival	
	HR(95% CI)	*P* value	HR(95% CI)	*P* value
**Superficial site (Subcutaneous, other soft tissue)**				
Head & Face & Neck	1.00 Reference		1.00 Reference	
Upper limb & Shoulder	0.814(0.556–1.192)	0.290	0.708(0.559–0.896)	0.004
Lower limb & Hip	1.149(0.832–1.585)	0.399	0.906(0.748–1.098)	0.314
Thorax & Breast	1.803(1.294–2.513)	<0.001	1.128(0.919–1.384)	0.249
Abdomen	3.170(2.241–4.484)	<0.001	2.210(1.775–2.752)	<0.001
Pelvis	2.097(1.461–3.011)	<0.001	1.678(1.343–2.096)	<0.001
Trunk	1.803(1.152–2.821)	0.010	1.313(0.977–1.765)	0.071
Overlap and Other	5.059(3.410–7.508)	<0.001	3.018(2.317–3.391)	<0.001
**Internal site (Includes bone or viscera** ***et al*** *.* **)**				
Digestive system	1.00 Reference		1.00 Reference	
Respiratory system	1.312(1.017–1.693)	0.037	1.121(0.924–1.359)	0.248
Reproductive system	1.113(0.814–1.521)	0.502	1.076(0.846–1.369)	0.551
Locomotor system	0.734(0.508–1.061)	0.100	0.900(0.696–1.165)	0.425
Urinary system	1.369(0.953–1967)	0.089	1.235(0.926–1.647)	0.150
Nervous system	0.962(0.567–1.633)	0.887	1.065(0.734–1.544)	0.740
Endocrine system	1.537(0.565–4.180)	0.400	0.946(0.419–2.136)	0.893
Circulatory system	2.297(1.149–3.827)	0.016	1.901(1.138–3.174)	0.014
Peritoneum& Spleen	1.370(1.050–1.789)	0.021	1.252(1.019–1.539)	0.033

The several important limitations that come with this study were acknowledged. Most importantly, the use of other treatment modalities is not recorded in the SEER database. Thus we could not identify the role of other treatment modalities, like radiotherapy or chemotherapy, in treatment for SCS. Besides, there are lack of information neither about surgery type nor resection margin status of the tumor. Similarly, it should be noted that some other important data specifically relevant to the tumor including TNM stage, AJCC stage, margin status, local or distant recurrence, lymphatic metastasis status, are either incomplete or absent. Additionally, we have to point out that the follow-up time in SEER is not even and long enough. However, this study is the first using such a large and comprehensive representative registry database to demonstrate the demographic features, clinic-pathologic characteristics, prognostic factors of spindle cell sarcoma.

In summary, it is definitely the largest data about SCS which came from SEER database. Despite its preliminary character, this study can clearly indicate the information on demographic features, distinctive clinicopathologic characteristics, tumor specific prognostic factors and treatment outcome by performing comprehensive analysis of the 3299 SCS cases from the database. The study does demonstrate that SCS mostly occurred during fifth to seventh decade of life in white people without gender specific. More importantly, we found that the ageå 64 years (≤64 years - as a ref), AJCC stage III (AJCC stage I - as a ref), SEER historic stage distant metastasized tumor (Localized tumor - as a ref) and primary tumor site in internal site (Tumor locate in superficial site - as a ref) were independent averse prognostic factors for SCS patients in DSS and OS. Despite the lack of the information about other treatment modalities (radiotherapy or chemotherapy), surgical resection shows the mainstay of treatment modality.

## Materials and Methods

The data extraction and statistical analysis were performed as described previously^19,20^. In brief, the data were extracted with International Classification of Diseases for Oncology codes 8801/3 for SCS from 1973 to 2017 by using official software SEER*Stat, version 8.3.4. Overall statistical analysis was performed by utilizing the software of the Statistical Package for Social Sciences, version 23.0, for Windows (SPSS, Chicago, IL) and survival tabs were generated by GraphPad Prism, version 5.01. The survival curves were generated by using the Kaplan-Meier method, and the survival difference was evaluated by performing the log-rank test. Adjusted hazard ratios (HRs) along with 95% confidence intervals (CIs) were calculated by using the Cox proportional hazards regression model. Differences in the numerical variables were assessed using the Student’s test or non-parametric Wilcoxon test. Categorical variables comparisons were evaluated by the chi square test or Fisher exact test. When the P value was <0.05, the difference was regarded as statistically significant. All statistical tests were two tailed.

### Ethical approval

This article does not contain any studies with human participants or animals performed by any of the authors.
